
JOURNAL INFORMATION
==============================
NLM Title Abbreviation: Gastroenterol Rep (Oxf)
Journal ID: Gastroenterol Rep (Oxf)
NLM Journal ID: 101620508
Journal ID: gastro

Gastroenterology Report

EISSN: 2052-0034
Publisher: Oxford University Press

ARTICLE INFORMATION
==============================
PMCID: PMC8677508
DOI: 10.1093/gastro/goab055
Article ID: goab055
Article version: 1
Subjects: Chinese Abstracts, AcademicSubjects/MED00260

Chinese Abstracts – Volume 9 Issue 6*

This article abstract was originally published in English. This translation has not been verified and should not be relied upon; it is provided for reference purposes only. The publishers have not checked this translation and accept no liability for completeness or accuracy of this translation or the use of this translation for whatever purpose. This translation may be incomplete and inaccurate in whole or in part. If you need to rely upon a translation of this abstract, a professional human translator should be engaged to supply an accurate translation of the original English. When referencing articles from this journal, please always refer to the original English version, rather than a translated equivalent

Publication date: 2021 Dec
Electronic publication date: 2021 Dec 16
Volume: 9
Issue: 6
First page: C1
Last page: C3
Copyright: © The Author(s) 2021. Published by Oxford University Press and Sixth Affiliated Hospital of Sun Yat-sen University
Copyright year: 2021
License: This is an Open Access article distributed under the terms of the Creative Commons Attribution License (https://creativecommons.org/licenses/by/4.0/), which permits unrestricted reuse, distribution, and reproduction in any medium, provided the original work is properly cited.
License URL: https://creativecommons.org/licenses/by/4.0/

Page count: 3

==============================

OXFORD

*Gastroenterology Report*, 9(6), 2021, 1–3

doi: 10.1093/gastro/goab055
Abstracts

ABSTRACTS

### Chinese Abstracts – Volume 9 Issue 6*

### Review（综述）

### One stomach, two subtypes of carcinoma—the differences between distal and proximal gastric cancer
### 一胃两癌——远端胃癌和近端胃癌的区别

胃癌是消化道最常见的恶性肿瘤之一, 严重威胁人类健康。在过去的十年, 我们基于胃癌的部位来判断其病理特征和预后, 将其分为近端胃癌和远端胃癌。本文就两类胃癌间流行病学、病因、细胞来源、病理特征、基因表达、分子标志物、临床表现、治疗、预后及预防等方面的差异作一综述, 为临床诊治提供指导和依据。

### Hepatorenal syndrome in acute-on-chronic liver failure with acute kidney injury: more questions requiring discussion
### 慢加急性肝衰竭患者出现肝肾综合征合并急性肾损伤：更多问题需要探讨

在肝硬化伴腹水的患者中, 肝肾综合征（HRS）是一种对液体扩容治疗无反应的特殊的肾前性功能障碍。慢加急性肝衰竭（ACLF）是一种临床综合征， 易发生多器官功能衰竭且早期病死率高。东西方医学界对于ACLF的定义存在差异。ACLF患者发生急性肾损伤（AKI）时多为器质性损伤， 导致在诊断HRS-AKI时存在诸多困惑。本文对ACLF患者中HRS-AKI的发病机制、诊断和治疗等方面的研究进展作一综述， 并着重讨论ACLF合并HRS-AKI的诊断中存在的一些争议问题, 如急性肝衰竭、肝癌、休克、脓毒血症和慢性肾脏疾病等。ACLF合并HRS-AKI的治疗有待进一步积极探索, 以改善其预后。

### Original article （论著）

### Genetic analysis of four consanguineous multiplex families with inflammatory bowel disease
### 四个炎症性肠病家系的遗传学分析

背景：家系研究支持炎症性肠病（IBD）的遗传易感性, 但已知的遗传变异只能部分解释该病的异质性。拥有多名患病个体的家系可能藏有某些罕见但强致病的变异。由于近亲繁殖导致的较长的纯合区域会增加个体出现纯合型失功能变异的风险。本研究旨在鉴定导致IBD的罕见纯合型遗传变异。

方法：招募4个拥有多名IBD患病成员的家系进行研究。采用纯合子定位法并辅以全基因组测序进行家系特异性分析。

结果：我们从一个德鲁士家系的多名克罗恩病成员中发现了单个纯合区域, 长度为2.6 Mb并包含*NOD2*基因。全外显子测序并没有在该区域内鉴定出任何潜在致病变异, 提示致病因素为非编码区变异。此外, 我们在这4个家系患病成员的自噬相关和自然免疫相关的基因中， 包括*LRRK1*、*WHAMM*、*DENND3*和*C5*, 发现了一些罕见的潜在致病的纯合型变异。

结论：本研究从IBD家系中检测出了一些罕见的潜在致病的纯合型变异。尽管没有进行统计学分析， 但本研究结果发现了一些值得进一步深入研究的基因或其核心区域。对于德鲁士克罗恩病患, *NOD2*基因的非编码区变异可能是重要致病因素。

### Comparing the clinical application values of the degree of ulcerative colitis burden of luminal inflammation (Dublin) score and ulcerative colitis endoscopic index of severity (UCEIS) in patients with ulcerative colitis
### 溃疡性结肠炎肠道炎症负担程度评分（Dublin）与溃疡性结肠炎内镜严重度指数（UCEIS）临床应用价值的比较

背景：内镜评估在溃疡性结肠炎（UC）的诊治中的重要意义已被广泛认可, 学者们已经建立了一些内镜评分系统。本研究对Mayo内镜亚评分（Mayo ES）、溃疡性结肠炎肠道炎症负担程度评分（DUBLIN）和溃疡性结肠炎内镜严重度指数（UCEIS）三种评分系统的临床应用价值进行了评估， 比较各个评分系统与UC疾病活动度的相关性， 及其对治疗反应和临床结局的预测效能。

方法：回顾性纳入2015年9月至2019年9月间两家医院的UC住院患者。采用Spearman秩相关分析来评估上述内镜评分系统与临床参数的线性关系。采用受试者操作特征曲线（ROC）来评估内镜评分系统对升阶梯治疗和1年再入院的预测价值。

结果：178例患者纳入研究， 其中82%系中重度结肠炎。48例（27%）患者接受了升阶梯治疗， 59例（33%）于1年内再入院。DUBLIN和UCEIS评分与临床参数的相关性高于Mayo ES。DUBLIN评分可以有效区分轻、中、重度结肠炎（均$P < 0.001$）。UCEIS评分对升阶梯治疗和1年再入院的预测能力最强, 曲线下面积分别为0.88和0.75。与UCEIS和DUBLIN评分相比, Mayo ES对升阶梯治疗（均$P < 0.001$）和1年再入院（$P < 0.001$; $P = 0.002$）的预测能力相对更低。UCEIS评分还能有效区分激素治疗有效与激素依赖或无效的患者（均$P < 0.001$）, 然而, Mayo ES和DUBLIN评分在上述三类患者中的差异无统计学意义（均$P > 0.05$）。

*This article abstract was originally published in English. This translation has not been verified and should not be relied upon; it is provided for reference purposes only. The publishers have not checked this translation and accept no liability for completeness or accuracy of this translation or the use of this translation for whatever purpose. This translation may be incomplete and inaccurate in whole or in part. If you need to rely upon a translation of this abstract, a professional human translator should be engaged to supply an accurate translation of the original English. When referencing articles from this journal, please always refer to the original English version, rather than a translated equivalent.
本文摘要最初是以英文发表。中文摘译未获认证，谨供参考。出版社没有对中文摘译进行核验，不负责译文的完整性和准确性，也不会将其用以其他目的。中文摘译可能会出现部分不完整或不准确的情况。如果你需要准确获悉摘要，最好能请一位专业译者针对英文原文进行翻译。阅读杂志文章，请参考英文原文，不推荐中文翻译。

结论：本研究显示，DUBLIN评分和UCEIS评分在评估UC患者疾病严重程度和预测治疗反应及临床结局方面，优于Mayo ES评分。

### Superiority of urgent vs early endoscopic hemostasis in patients with upper gastrointestinal bleeding with high-risk stigmata
### 对于高危上消化道出血患者，急诊内镜与早期内镜止血孰优孰劣？

背景：指南建议所有上消化道出血（UGIB）患者在24 h内接受内镜检查，但尚不清楚是否存在能从急诊干预中获益的患者。本研究旨在评估内镜止血的疗效，以及急诊内镜对于具有高危出血灶（HRS）的UGIB患者病死率的影响。

方法：连续纳入日本3家医院的疑似UGIB患者，并按要求在24 h内对患者进行内镜检查。主要结局指标是30天病死率。通过多因素分析评估内镜止血和内镜检查时机（急诊内镜≤ 6 h；早期内镜> 6 h）对病死率的影响，调整的致混淆因素包括患者年龄、收缩压、心率、血红蛋白水平、肌酐水平以及静脉曲张性出血。同时，通过1:1倾向性评分匹配进行敏感性分析。

结果：1966例患者纳入分析，其中886例存在HRS，35例（3.95%）死亡。急诊内镜（$n = 769$）的中位检查时间为3.0 h（IQR：2.0 – 4.0 h）；早期内镜（$n = 117$）中位检查时间为 12.0 h（IQR：8.5 – 19.0 h）。多因素分析显示，成功内镜止血（OR：0.22, 95% CI：0.09 – 0.52, $P = 0.0006$）和急诊内镜干预（OR：0.37；95% CI：0.16 – 0.87，$P = 0.023$）可以显著降低病死率。经倾向性评分匹配调整后，115对患者纳入分析，结果显示，相较于早期内镜，急诊内镜干预的病死率显著较低（2.61% vs 7.83%, $P < 0.001$）。

结论：具有HRS的UGIB患者，可以从内镜止血中获益，而且急诊内镜较早期内镜能进一步降低病死率。制定一个能够预测HRS的评分系统来指导治疗选择是非常重要的。

### Classification and genetic counselling for a novel splicing mutation of the *MLH1* intron associated with lynch syndrome in colorectal cancer
### 与林奇综合征结直肠癌相关的*MLH1*一个新的内含子剪接突变的分类及遗传咨询

背景：林奇综合征相关癌症是由错配修复基因胚系致病性突变引起的。林奇综合征筛查的主要挑战是对诊断性检测中所发现变异的解释。本研究旨在对曾被报道为意义不明的变异体*MLH1* c.1989 + 5G>A进行分类、描述其临床表型和特征，并进行详细的遗传咨询。

方法：查阅我院林奇综合征患者基因检测数据库，并进一步通过二代测序来鉴定*MLH1* c.1989 + 5G>A新的变异体。使用免疫组织化学染色评估肿瘤组织中MLH1和PMS2蛋白的表达。通过Alamut软件进行的计算机分析预测*MLH1* c.1989 + 5G >A变异体的功能；并采用逆转录PCR和全血RNA测序分析该突变的功能意义。

结果：在疑似林奇综合征家系的肿瘤患者中，均患有晚期结直肠癌，但预后良好。我们发现*MLH1* c.1989 + 5G>A变异体会导致异常剪接，最终造成肿瘤细胞核中MLH1和PMS2蛋白表达缺失。在*MLH1* c.1989 + 5G>A的携带者中检测到一个异常的转录本，并证实其发生了*MLH1* 17号外显子的跳跃。

结论：在癌症家系中检测到的*MLH1* c.1989 + 5G>A被鉴定为林奇综合征患者的致病性变异体。加强基因检测丰富了林奇综合征的突变谱，密切监测可能有助于疑似林奇综合征患者得到明确的早期诊断。

### Elevated peripheral blood neutrophil-to-lymphocyte ratio is associated with an immunosuppressive tumour microenvironment and decreased benefit of PD-1 antibody in advanced gastric cancer
### 外周血中性粒细胞/淋巴细胞比值升高导致进展期胃癌患者的肿瘤微环境免疫抑制并降低PD-1抗体的治疗获益

背景：虽然PD-1单抗在胃癌治疗中取得了里程碑式的进展，但由于其疗效有限，加之潜在的毒性，因此并不适用于所有晚期胃癌患者。我们迫切需要找到能够预测PD-1单抗治疗获益人群的生物标志物。本研究旨在从系统炎症状态和肿瘤微环境的角度，评估进展期胃癌患者外周血炎症标志物的疗效预测价值及预后价值。

方法：本研究从一项针对特瑞普利单抗单药用于进展期胃癌患者安全性及有效性评估的前瞻性临床研究中纳入了58例患者。分析患者的临床病理特征、血液学炎性指标和治疗疗效；并通过转录组测序评估肿瘤组织中的免疫细胞浸润和相关基因表达情况。

结果：该队列中位随访时间为4.5个月，中位无进展生存期为1.9个月，中位总生存期4.8个月，客观缓解率为12.1%，疾病控制率为39.7%。基线外周血中性粒细胞/淋巴细胞比值（bNLR）>2.7和首次给药后2周bNLR动态变化（dNLR）>1.5倍是不良预后因素，且bNLR或dNLR高的患者疾病控制率相对较差（25.8% vs 59.1%, $P = 0.023$；15.8% vs 54.6%，$P = 0.008$）。多因素分析中证实，bNLR和肿瘤突变负荷是总生存的独立预后因素。转录组测序分析结果显示，基线bNLR高的患者，其肿瘤组织具有更多的中性粒细胞浸润和更高的NLR；差异基因分析显示，bNLR高的患者中性粒细胞招募相关基因和炎性细胞因子明显富集。

结论：我们的研究表明，NLR可作为预测晚期胃癌患者预后及抗PD-1治疗效果的潜在生物标记物，用于临床病例筛选和预后判断。此外，bNLR增高可以反映出肿瘤组织中的NLR失衡以及肿瘤微环境的免疫抑制状态。

### Clinical characteristics and survival analysis of colorectal cancer in China: a retrospective cohort study with 13,328 patients from Southern China
### 中国结直肠癌的临床特征和生存分析：中国南方13,328例患者的回顾性队列研究

背景：结直肠癌是中国最常见的癌症之一，然而，关于中国结直肠癌患者临床特征和生存的详细数据尚不充分。本研究旨在探讨中国南方结直肠癌患者的流行病学特征和潜在的预后因素。

方法：纳入1994—2019年在中山大学第一附属医院和第六附属医院就诊的原发性结直肠癌患者，收集患者的临床特征和生存结局。Kaplan-Meier方法用于估计总生存期和无进展生存期，Cox比例风险回归模型用于评估预后危险因素。

结果：共计13,328例结直肠癌患者纳入研究，其中男性占60.1%，平均年龄61.3岁，53.5%为结肠癌，14.0%被诊断为IV期。全组患者中位总生存期189个月，3年和5年总生存率分别为79.90%和71.50%；无进展生存期149个月，3年和5年无进展生存率分别为70.30%和63.90%。低/未分化癌患者中有428例（14.0%）出现复发。在III期和IV期患者中，接受化疗的患者中位无进展生存期显著高于未化疗者（III期：147 vs 62个月，$P < 0.001$；IV期：14 vs 9.5个月，$P < 0.001$）。

结论：本研究呈现了中国南方结直肠癌患者的临床特征。性别、年龄、家族史、肿瘤发生部位、分化程度、T分期、N分期、M分期、临床分期、手术方式及手术切缘均为结直肠癌患者的独立预后因素。

### Robotic total gastrectomy for carcinoma in the remnant stomach: a comparison with laparoscopic total gastrectomy
### 机器人全胃切除术治疗残胃癌：与腹腔镜全胃切除术的比较

目的：全胃切除术治疗残胃癌技术难度大。相较于腹腔镜手术，机器人手术治疗残胃癌的优劣尚不明确。本研究旨在比较机器人全胃切除术（RTG）与腹腔镜全胃切除术（LTG）治疗残胃癌的疗效和安全性。

方法：采用回顾性队列研究的方法，纳入2006年5月至2019年10月期间在重庆西南医院接受RTG或LTG治疗的残胃癌患者。比较两组患者的手术结果、术后并发症和生存情况。

结果：与LTG组相比，RTG组患者有效手术时间相当（272.0 vs 297.9 min，$P = 0.170$），术中出血量较少（229.2 vs 288.8 mL，$P = 0.031$），住院总花费更高（105,967.2 vs 81,629.5元，$P < 0.001$）。中转开腹率、术后首次排气时间、术后首次进食时间、术后住院时间、术后并发症发生率、R0切除率及淋巴结清扫数目差异等方面两组差异

均无统计学意义（均$P > 0.05$）。两组患者3年无病生存率（65.5% vs 57.5%, $P = 0.918$）和3年总体生存率（69.0% vs 60.0%, $P = 0.850$）的差异亦无统计学意义。

结论：RTG治疗残胃癌安全可行, 可作为残胃癌一种治疗选择。

### Avoidance of urinary drainage during perioperative period of open elective colonic resection within enhanced recovery after surgery programme

### 在加速康复外科理念下, 择期开放结肠切除术围手术期不必放置尿管

背景：放置尿管是腹部手术一项传统的围手术期管理措施, 而对围手术期置管的优化则是加速康复外科（ERAS）的重要内容之一。本研究旨在调查ERAS理念下开放结肠切除术后放置尿管的危险因素, 并评估围手术期不置尿管的可行性。

方法：2014年7月至2018年5月间接收开放手术的110例结肠癌患者纳入研究。所有患者均按我们中心的ERAS方案进行围手术期管理。收集人口统计资料、临床病理特征和围手术期结局进行回顾性分析。

结果：68（61.8%）例患者围手术期未置尿管, 30例（27.3%）术中放置尿管, 12例（10.9%）因术后尿潴留放置尿管。尽管术中放置尿管者术后尿潴留发生率更低[0% (0/30) vs 15% (12/80), $P = 0.034$]。但多因素分析显示, 术中置尿管并不是术后尿潴留的独立保护因素；前列腺病史和体质指数则是术后尿潴留的独立影响因素。6例患者出现术后尿路感染, 其中2例为术中置尿管者, 另4例伴发术后尿潴留因而需要放置尿管。

结论：遵循ERAS方案, 择期开放结肠切除术围手术期不置尿管是可行的。肥胖和前列腺病史是术后尿潴留的危险因素。

### Brief report（短篇报道）

### Higher risk of neoplastic progression of barrett's esophagus in patients with systemic sclerosis

### 合并系统性硬化症的barrett食管患者具有更高的瘤变风险

### Treatment of colonic varices with a superior mesenteric venous stent: a case report describing a unique approach

### 采用肠系膜上静脉支架治疗结肠静脉曲张：独特的技术

### Best practices in teaching endoscopy according to a delphi survey of gastroenterology trainees

### 根据消化科规培医师的delphi调查制定的最佳教学内镜实践

### First preclinical experience with the newly developed EDGE SP1000 single-port robotic surgical system-assisted transanal total mesorectal excision

### 最新研发的EDGE SP1000单孔机器人手术系统辅助经肛全直肠系膜切除术的临床前研究

### Commentary（评论）

### Tackling clinical complexity: How inpatient subspecialty gastroenterology services enhance patient care

### 临床复杂性的应对策略：如何通过消化病房亚专科服务提升患者医护水平
